# Genetic Parameters of Serum Total Protein Concentration Measured with a Brix Refractometer in Holstein Newborn Calves and Fresh Cows

**DOI:** 10.3390/ani13030366

**Published:** 2023-01-20

**Authors:** Aikaterini Soufleri, Georgios Banos, Nikolaos Panousis, Alexandros Kougioumtzis, Vangelis Tsiamadis, Georgios Arsenos, Georgios E. Valergakis

**Affiliations:** 1 Laboratory of Animal Husbandry, Faculty of Veterinary Medicine, School of Health Sciences, Aristotle University of Thessaloniki, P.O. Box 393, 54124 Thessaloniki, Greece; 2 Scotland’s Rural College, Roslin Institute Building, Easter Bush, Midlothian EH25 9RG, Scotland, UK; 3 Clinic of Farm Animals, Faculty of Veterinary Medicine, School of Health Sciences, Aristotle University of Thessaloniki, 54124 Thessaloniki, Greece

**Keywords:** dairy cows, newborn calves, total proteins, refractometer

## Abstract

**Simple Summary:**

The adequate passive transfer of immunity, which is estimated indirectly and rapidly through measuring serum total proteins, is vital for newborn calves. At the same time, cows’ serum total proteins concentration is considered an indicative parameter of their health and metabolic status. In the present study, the genetic parameters of Holstein dairy cow and calf total proteins concentration measured by a digital Brix refractometer were studied. Heritability estimates of the traits suggest that genetic selection would be feasible and useful. In addition, results offer interesting perspectives regarding calf and cow health and, therefore, herd sustainability. The high positive genetic correlation between calf serum total proteins and colostrum total solids, is a very important finding and allows us to perform genetic selection for both traits simultaneously by collecting a single phenotype.

**Abstract:**

The objective was to estimate the genetic parameters of serum total protein concentration in newborn calves (calfSTP) and post parturient dairy cows (cowSTP). The study included 1013 calves and 989 cows from 10 dairy farms. Calf blood samples were collected 24–48 h after parturition while cow blood and colostrum samples were collected in the first 24 h after calving. Blood serum total protein and colostrum total solids content were determined using a Brix refractometer. Chemical analysis of colostrum was performed with Milkoscan. Univariate mixed linear models were used to estimate the heritability of calfSTP and cowSTP and their genetic and phenotypic correlations with colostrum traits. The heritability estimates of calfSTP and cowSTP were 0.21 and 0.20 (*p* < 0.05), respectively. Strong genetic correlations (r > 0.90) were detected between calfSTP and colostrum total solids and protein content (*p* < 0.05). Corresponding phenotypic correlations were 0.31–0.33 (*p* < 0.05). No genetic or phenotypic correlations were detected with colostrum fat content while the respective correlations with lactose were negative (−0.82 and −0.19, *p* < 0.05). No genetic correlations were detected between cowSTP and colostrum traits and only a low negative phenotypic one with lactose was detected. The results confirm that genetic selection aiming to improve the passive transfer of immunity in newborn calves and general fresh cow health would be feasible.

## 1. Introduction

The transfer of passive immunity is vital, both for the short- and long-term health and productivity of dairy calves. Unsuccessful transfer has been associated with morbidity and mortality rates [1,2] and increased financial losses [3].

Cattle placenta is of the epitheliochorial type which is impermeable to immunoglobulins in utero [4]. Calves are born agammaglobulinemic, that is, without circulating antibodies [1,5,6]. Since their immune system is immature and naïve at birth, they are not capable of producing enough of their own antibodies until the age of 3 to 6 weeks [7]. Therefore, they are directly dependent on the ingestion and absorption of immunoglobulins contained in colostrum [8], which contains additional substances related to calf health such as IL-6 [9]). Consequently, the prompt administration of adequate quantities of high-quality colostrum to newborn calves constitutes a management practice integral to the successful transfer of passive immunity and, by extension, it is essential for their survival, health, and future production [10].

The assessment of immunity transfer to newborn calves is best accomplished by measuring the concentration of immunoglobulins G (IgG) in blood serum using radial immunodiffusion (RID) and is considered adequate when the IgG concentration is higher than 10 mg/mL during its first days of life [1,8,11]. This direct method is considered as the golden standard but requires a laboratory set and is expensive and time-consuming. Currently, rapid indirect methods are used for the determination of the successful transfer of immunity such as the measurement of calf serum total proteins (STP, in g/dL), either using an automated analyzer or a graded refractometer [12,13,14]. Alternatively, the measurement of calf serum Brix value (%), which is well correlated with STP concentration [15,16,17], either with optical or digital refractometers can be used.

Several management and environmental factors that affect the transfer of passive immunity have been investigated thoroughly and reported in published studies. Poor quality [2,7,18] and low quantity of colostrum fed [18,19], an extensive time interval from birth to first colostrum administration [6,19], high bacterial contamination [20,21,22], postnatal respiratory acidosis [23] or hypoxia in calves [24], and the exposure of calves to high temperatures [25] can lead to low absorption of IgG and an insufficient transfer of passive immunity. Still, even when these environmental factors have been considered, a relatively large number of calves fail to acquire passive immunity, and this could be attributed to genetics.

Estimations of the genetic parameters for the transfer of passive immunity in calves have been reported. Genetic studies so far have focused on heritability estimates of calf STP (g/dL) and IgG concentrations. Heritability estimates reported for calf serum IgG concentration was high (0.52–0.56) in some studies [26,27] and moderate (0.15–0.25) [28,29,30] or even low (0.03) in others [31]. Reported heritability estimates for calf STP concentration (g/dL) range from low to moderate, 0.06 to 0.36, respectively [30,32,33]. However, only Johnston et al. [28] estimated the heritability of calf STP concentration measured in a Brix scale and reported a low value of 0.05. Considering the available literature, there are not any studies of genetic and phenotypic correlations between the passive transfer of immunity and colostrum quality traits in cows. Moreover, animal phenotype collection with a Brix refractometer is much easier, faster, and less expensive. Both the former and the latter were the main incentives behind the present study.

Dairy cow STP concentration is considered a parameter indicative of health and metabolic status [34,35,36]. Both the albumin and the globulin fractions have several functions [37] and fluctuations in their concentrations that could signify (or imply) pathological or physiological processes [36]. Albumin is synthesized in the liver and serves as a transport for many substances [38]. Low levels could be a marker of liver malfunction owing to inflammatory conditions [36,39]. Globulins are a heterogeneous pack of proteins which consists of antibodies and other inflammatory molecules, carriers of proteins and lipids, vitamins, and hormones [36]. Chorfi et al. [40] propose that globulin concentration could provide information regarding the animal’s immune response. In daily veterinary practice, the albumin to globulin ratio (A:G) has substantial interest as it is a useful tool to identify endometritis and dysproteinemia [41,42,43] and it has been suggested as an indicator for the determination of cow immune status [44].

In previous studies, the concentration of cow STP has been assessed using automated analyzers [36,37,38]. Several individual (stage of lactation, parity, and health) and environmental factors (season, dairy production system, and nutrition) that affect the concentration of cow STP have been reported in published studies and different reference values have been suggested according to season [35], age [35,45], and stage of lactation [35,38]. Cecchinato et al. [37] reported heritability estimates for cow STP, albumins, globulins, A:G ratio (estimated heritabilities being 0.20, 0.13, 0.20, and 0.21, respectively), and the genetic correlations between them. Studies on STP concentration in cows based on a digital Brix refractometer have never been published and consequently no genetic studies have either. In fact, the correlation between cow STP measured with an automated analyzer and that estimated with a Brix refractometer has never been explored before. Moreover, genetic and phenotypic correlations between cow STP and colostrum traits are missing in the literature, as well.

Therefore, the objectives of this study were: (a) to estimate the heritability of newborn calf STP concentration using by a Brix refractometer; (b) to find the correlation between cow STP measured with an automated analyzer and with a Brix refractometer and estimate the heritability of fresh cow STP concentration measured by a Brix refractometer; and (c) to investigate genetic and phenotypic correlations between calf and cow STP concentrations and between calf/cow STP concentrations and colostrum traits.

## 2. Materials and Methods

This research was conducted in compliance with institutional guidelines and was approved by the Research Committee of the Aristotle University of Thessaloniki, Greece (Approval number: 50/17-02-2015). All farmers gave informed consent for their calves and cows to be included in the study.

### 2.1. Animals and Management

The study included a total of 1020 Holstein newborn calves and their dams. These were kept in 10 commercial dairy farms in the region of Central Macedonia (Northern Greece) with herd size ranging from 90 to 300 cows. Sample collection and data recording lasted 19 months. Dry cows were housed in straw-yard sheds; on six farms they were kept in a single group while on four farms far-off and close-up groups were formed. On all farms, dry cows were offered total mixed rations comprising of corn silage, wheat straw, soybean meal and -a mineral/vitamin supplement. Rations were, in all cases, formulated to meet or exceed NRC (2001) recommendations [46] for net energy lactation (NEL) and metabolizable protein (MP).

### 2.2. Clinical Examination and Collection of Blood Samples from Calves and Cows

All calves were viable and full term, received maternal colostrum, and were clinically examined by a qualified veterinarian, 24 to 48 h after birth [8]. Calves exhibiting any sign of disease were excluded from the study. At the same time, their body weight was estimated using a heart girth tape. Quantity of colostrum meal fed to each calf and time interval between birth and colostrum administration were recorded.

Calves were blood sampled between 24 to 48 h post-calving and STP in calf serum were measured within 3 h from sampling. Blood samples, in all herds, were collected between 0800 h and 1000 h. Blood sampling was performed by jugular veni-puncture into 10 mL vacuum plastic tubes without anti-coagulant (BD Vacutainer, Plymouth, UK). Time interval between birth and blood sampling was recorded. No colostrum from other cows, fresh or frozen, was offered to calves included in the study. Calves were separated from their dams within 30 min from birth. Calves suspected to have suckled their dams or other cows were excluded from the study.

At the day of calving, all cows were subjected to a complete clinical examination and those with any disease sign or producing abnormal colostrum (containing blood or clots, watery appearance) were not included in the study. Regarding the calving ease, 80% of cows had score 1, 13% score 2, and 7% score 3. Cows that had undergone caesarean section were not included in the study.

Using proper cow restraint, blood samples were collected between 0800 h and 1000 h by coccygeal veni-puncture into 10 mL vacuum glass tubes without anti-coagulant (BD Vacutainer, Plymouth, UK); 305-d milk yield of previous lactation and dry-period length were recorded.

All samples from calves and cows were placed in a cooler and transported to the Laboratory of Animal Husbandry of the Aristotle University of Thessaloniki Faculty of Veterinary Medicine for handling and analysis.

### 2.3. Determination of STP in Calves and Cows

Blood samples were centrifuged [4000× *g* for 15 min at room temperature (21 °C)] immediately upon arrival at the Laboratory. Serum total protein (STP) concentrations (%Brix) were measured using a digital Brix refractometer (PAL-1, Atago, Japan) placing a quantity of 0.3 mL on the prism surface. The instrument has a measurement range from 0 to 53%, a high accuracy of ±0.1% and is independent of ambient temperature (0–99 °C). Distilled water was used for the calibration before each measurement. All measurements were performed twice, recorded, and subsequently averaged.

### 2.4. Correlation between Cow STP Measured with an Automated Analyzer and a Brix Refractometer

An independent dataset comprising of 245 cow samples, collected from 3 different farms, between 21 days prior to and 30 days after calving, was used.

### 2.5. Colostrum Samples

The collection of colostrum samples, on-farm determination of colostrum quality, and laboratory determination of colostrum composition are described extensively in Soufleri et al. [47]. Regarding the on-farm determination of colostrum quality, 83% of the samples presented a Brix measurement more than 22% and 60% of the samples more than 25%. The protocol for the determination of colostrum fat, protein and lactose content with MilkoScan is described in Supplementary Material S1.

### 2.6. Final Dataset

Seven calves and thirty-one cows were excluded from the study due to health issues and sample suitability. The final dataset included 1013 calves and 989 cows. For cows, the distribution of cows across parities was 346, 291, 166, and 186 for parities 1, 2, 3, and ≥4, respectively.

Calf STP (%Brix value) data were matched with farm records including the identification and parity number of dams, date of calf birth, calving ease, calf gender and body weight, quality and quantity of colostrum fed, and time intervals between birth and colostrum administration, and blood sampling.

Cow STP (%Brix value) were matched with farm records including dry period length (for cows with parity ≥ 2), quality and quantity of colostrum produced, milk yield of previous 305-d lactation (for cows with parity ≥ 2), age at calving (in months) and parity number, and date of last calving.

Quality traits of the colostrum fed to calves of study were total solids, protein, fat, and lactose contents. Descriptive statistics of colostrum administration are presented in Table 1.

### 2.7. Pedigree

As available pedigree information, for all healthy 1013 calves and 989 cows of the present study, spanned up to seven ancestral generations, 6368 animals were included in the analysis.

### 2.8. Statistical Analysis

The correlation between cow STP measured with an automated analyzer and a Brix refractometer was estimated with bivariate analysis (correlation coefficient by Pearson). Statistical analysis was performed using the IBM SPSS software (IBM Corp. Released 2015, IBM SPSS Statistics for Windows, Version 23.0. IBM Corp, Armonk, NY, USA).

Traits (cow and calf STP) were analyzed using the following mixed model:

(1) Y=Xb+Zu+e

where:

Y is the trait (dependent variable);

b is the vector of fixed effects;

u is the vector of random effects (animal additive genetic effect~N (0, Aς_a_^2^), where A was the pedigree relationship matrix);

e is the vector of random residual effects ~N (0, Iσ_r_^2^), where I was the identity matrix;

X,Z are incidence matrices linking the fixed and random effects, respectively, with the observations.

For the calf STP trait, fixed effects tested were year-calendar season of birth, calf gender and body weight, quality and quantity of colostrum fed, time interval between birth and colostrum administration, calving ease, and time interval between birth and blood sampling.

Colostrum quantity fed was included in Model (1) as a covariate. Season of birth was categorized as follows: winter (December, January, February), spring (March, April, May), summer (June, July, August), and autumn (September, October, November).

For the cow STP trait, fixed effects tested were number of parity (as 1, 2, 3, and ≥4), year-calendar season of calving (as in the calf STP analysis), length of dry period, and previous lactation 305-d milk yield. The latter two were included in the model as covariates and for cows with parity ≥ 2, only.

The statistical significance (*p* < 0.05) of all fixed effects above was confirmed in preliminary analyses.

Estimates of variance components of the random effects in Model (1) were used to calculate heritability for the trait, using the following equation:

$$h^{2}=\frac{\sigma_{\alpha }^{2}}{\sigma_{p}^{2}}$$

where

$h^{2}$ = the heritability estimate;

$\sigma_{\alpha }^{2}$ = the additive genetic effect variance estimate;

$\sigma_{p}^{2}$ = the phenotypic variance estimate calculated as the sum of $\sigma_{\alpha }^{2}$ and the residual effect variance.

Furthermore, in a series of bivariate statistical analyses based on Model (1), genetic and phenotypic correlations between cow and calf STP, and between STP and colostrum traits were derived from co-variance component estimates using the following equation:

$$r_{\left(\alpha ,p\right)}=\frac{{\mathrm{Cov}}_{\left(\alpha ,p\right)}\left(Χ,\;\Upsilon \right)}{\sqrt{\sigma_{\left(\alpha ,p\right)Χ}^{2}\times {\;\sigma }_{\left(\alpha ,p\right)\Upsilon }^{2}}}$$

where

${\mathrm{Cov}}_{\left(\alpha ,p\right)}\left(Χ,\;\Upsilon \right)$ = the additive genetic $\left({\mathrm{Cov}}_{\alpha }\right)$ or phenotypic $\left({\mathrm{Cov}}_{p}\right)$ co-variance of traits X and Y and $\sigma_{\left(\alpha ,p\right)Χ}^{2}$ and $\sigma_{\left(\alpha ,p\right)\Upsilon }^{2}$ are the respective genetic $\sigma_{\alpha }^{2}$ and phenotypic $\sigma_{p}^{2}$ variances.

Significance of the random effects in Model (1) were assessed with a likelihood ratio test and significance of the derived parameters (heritability and correlations) was assessed with a *t*-test.

All analyses were conducted with the statistical software package ASREML [48].

## 3. Results

The equation describing the relationship between STP measured with an automated analyzer and the estimation of a Brix digital refractometer is as follows: STP (g/dL) = 0.4235 × Brix value (%) + 2.508 (r = 0.60, *p* < 0.05).

Descriptive statistics of the studied traits are shown in Table 2. They were normally distributed (Skewness and Kurtosis). Variability of both calf and cow STP concentration was relatively low compared with the colostrum traits.

In Table 3, variance components and heritability estimates for STP traits are presented. Genetic variance and heritability estimates were moderate and statistically significant (*p* < 0.05) for both calf and cow traits.

The genetic and phenotypic correlations between calf and cow STP were −0.20 (0.30) and −0.02 (0.04) and were statistically not different from zero (*p* > 0.05). Phenotypic and genetic correlations between STP and the colostrum traits are presented in Table 4. Calf STP was genetically strongly correlated with all the traits of the colostrum fed to the calves except for fat content, positively with total solids and protein content (0.94 and 0.99, respectively), and negatively with lactose content (−0.82). On the contrary, cow STP was not genetically correlated with the traits of the colostrum retrieved from them. Calf STP was phenotypically moderately correlated with all the traits of the colostrum fed to the calves except for fat content, positively with total solids and protein content (0.33 and 0.31, respectively), and negatively with lactose content (−0.19). Cow STP was phenotypically negatively correlated with colostrum lactose content (−0.10) only.

## 4. Discussion

The objective of the present study was to estimate the genetic parameters of calf and cow STP concentrations measured by a Brix refractometer. A large data set of approximately 1000 Holstein cows and their calves was used in this study. To the best of our knowledge, heritability estimates of cow STP concentration and genetic and phenotypic correlations of calf and cow STP with colostrum traits are presented here for the first time.

The mean Brix value of calf STP concentration (8.7%) in the present study is slightly lower than that reported by Urie et al. [49] for heifer calves in the United States (9.2%) and very similar (8.9%) to that reported by Johnston et al. [28] for calves in Ireland. The moderate heritability estimate reported here (0.21) is in contrast with the low one (0.05) of the study of Johnston et al. [30], which so far has been the only one in the literature reporting heritability of Brix values. Nevertheless, direct comparisons between the two studies are not feasible since the Irish study included calves from several different breeds together with crossbreds, whereas purebred calves were used in the present study.

Phenotype collection on individual animals using the Brix refractometer is much simpler, faster, and cheaper compared with measuring IgG concentrations. This would facilitate the all too important data recording and collection step in farm management and genetic improvement programs. Of equal importance is the strong positive genetic correlation (0.99) of calf STP with colostrum total solids, the main practical colostrum quality trait. Duru et al. [33] also reported a very similar, highly positive, genetic correlation (0.98) between colostrum total solids and calf STP (g/dL), measured with a graded refractometer. This result practically means that we can carry out genetic selection for both traits simultaneously by collecting a single phenotype.

Furthermore, a significant, highly positive, genetic correlation was detected between calf serum Brix values and colostrum protein content and a significant, highly negative, genetic correlation between calf serum Brix values and colostrum lactose content. These findings are reported for the first time. Considering their respective correlations with colostrum total solids content [47] and the relationship of the latter with calf serum Brix values, these results are desirable and not surprising.

No significant correlations, genetic or phenotypic, were detected between calf STP and colostrum fat content. This was rather expected as no association had been previously found between colostrum total solids content and fat content [47]. There are no other relevant references in the literature. Colostrum fat content is relatively high [47] and it is essential for thermogenesis and the maintenance of body temperature of newborn calves. If our findings are confirmed in future studies, the lack of any correlation between calf STP and colostrum fat content would simplify genetic selection programs. A synthetic index could be potentially developed to combine all desirable nutritional colostrum traits and the transfer of passive immunity.

Moreover, the positive phenotypic correlation between calf serum STP and colostrum total solids is similar with that previously reported by Duru et al. [33], although in the latter study STP concentration was measured with a refractometer in g/dL. Combined with the positive phenotypic correlation between calf STP and colostrum protein content and the negative correlation with colostrum lactose content, this result suggests that the ingestion of higher colostrum quality by calves leads to the successful transfer of passive immunity.

Our results combined with those of most other studies measuring calf serum IgG and STP concentrations, corroborate the feasibility of genetic selection for the improvement of the passive transfer of immunity, thereby promoting calf health and welfare and herd profitability. Worldwide, breeding programs include traits with similar heritabilities, such as milk yield (h^2^ = 0.20–0.50) [50,51,52] and milk somatic cell count (h^2^ = 0.03–0.11) [53,54], and genetic selection has been successful. Certainly, genetic correlations among calf STP trait and other economically important cattle traits should be investigated before its inclusion in any selection index.

If only one trait were to be selected for recording, the choice between calf blood samples and colostrum samples would depend on applicability under field conditions; both approaches would produce the same result. The determination of STP concentration on farm conditions requires the combination of a centrifuge with a Brix refractometer. On the other hand, colostrum total solids evaluation requires a Brix refractometer only. Moreover, the latter tends to become a common management practice on most dairy farms. Of course, a specific protocol must be followed in order to acquire a credible phenotype, but farmers are expected to apply such a protocol since the immediate benefits on calf health are obvious. In either case, the refractometer offers a low-cost solution.

The correlation found between cow STP measured with an automated analyzer and STP estimation using a digital Brix refractometer was moderate. There is no published research addressing this issue. In calves, the correlations between calf STP measured by a refractometer in g/dL and STP measured with a refractometer in a Brix value range from 0.91 to 1.00 [15,55]. Nevertheless, the studies cannot be comparable as they use different type of refractometers and not an automated analyzer. There is no published study correlating Brix and analyzer measurements, either. In our study, the correlation was certainly not very high, but it was statistically significant (*p* < 0.05) and for the collection of phenotypes, specialized equipment was not needed.

Cow total protein values calculated with the equation developed in the present study ranged from 56.0 g/L to 95.1 g/L with a mean of 73.5 g/L; there are no other relevant references in the scientific literature. Concentrations of STP in cows measured with automated analyzers have been previously reported as values ranging between 67.80 g/L [45] and 75.23 g/L [37] with a low variability of 7% to 16%. Peek and Divers [56] reported a range of 72.00 to 90.00 g/L. It must be noted here that the genetic analysis was performed with the Brix values derived from the digital refractometer.

A moderate heritability (0.20) of cow serum Brix value was estimated in the present study; such references are missing from the literature. On the other hand, both Peterson et al. [57] and Cecchinato et al. [37] reported a similar moderate heritability (0.20) of cow STP (g/L) measured by an automated analyzer. According to Piccione et al. [38], gestation and lactation stages affect cow STP. Therefore, a single cow measurement is not indicative of changes during the transition from late gestation to early lactation and more measurements are needed to properly describe the transfer dynamics between cow serum and colostrum and the associations between them. More and extensive research is needed on this issue.

In the present study, no genetic correlations were detected between cow serum Brix value and colostrum traits. However, a low negative phenotypic correlation with colostrum lactose content was detected. There are no other relevant reports in the literature.

Further research is needed to confirm our findings regarding the genetic parameters of cow serum Brix value, which may provide useful information on cow health post calving and is associated with her metabolic status. The trait appears heritable and genetic selection could be feasible and beneficial. The use of a single measurement in the present study is probably not enough to capture the association of cow serum Brix value and colostrum traits and maybe a second measurement, for instance one week pre-calving, is needed. The lack of any correlation between cow and calf serum Brix values corroborates the lack of statistically significant genetic correlations between cow serum Brix and colostrum quality traits, since calf STP is associated with the quality and quantity of colostrum ingested.

## 5. Conclusions

The genetic variance and heritability estimates of calf serum STP, which offers an indirect assessment of the passive transfer of immunity based on Brix values, were statistically significant and, consequently, this trait could be improved with genetic selection. Considering the significant genetic correlations of this calf trait with maternal colostrum quality, significant genetic progress could be performed by the selection of cows with high-quality colostrum, whose phenotypes could be collected easily and with low cost. Moreover, cow STP concentration, estimated with a Brix refractometer, is also heritable and if this trait proves to be concretely correlated with cow health, could also be considered in genetic improvement. More research is needed to corroborate these results. The identification of genomic regions associated with the studied traits and their correlation with other cow and calf traits should be investigated in future studies.

## Figures and Tables

**Table 1:** Descriptive statistics of colostrum administration practices.

Trait (Unit of Measurement)	Mean	SD	Median	Min	Max
Interval between birth of calf and colostrum administration (min)	234	183	180	15	780
Quantity of colostrum fed (L)	2.4	0.9	2.5	0.5	6.0
Quality of colostrum fed (%Brix value)	25.8	4.6	25.9	10.7	41.4

**Table 2:** Descriptive statistics of cow and calf serum protein concentration (Brix value) and colostrum traits (%).

Trait (Unit of Measurement)	Mean	SD	Median	Min	Max
Calf serum total proteins	8.7	1.0	8.7	6.4	13.0
Cow serum total proteins	9.5	0.7	9.5	7.3	12.4
Colostrum total solids	25.8	4.7	25.9	10.7	41.4
Colostrum protein	17.8	3.9	17.8	4.8	30.4
Colostrum fat	6.4	3.3	5.9	0.1	18.2
Colostrum lactose	2.1	0.7	2.2	0.1	5.0

**Table 3:** Variance component and heritability estimates of cow and calf serum total proteins (%Brix value).

Trait (Unit of Measurement)	σ_p_^2^	σ_a_^2^	σ_r_^2^	h^2^
Calf serum total proteins	0.90 (±0.04) *	0.18 (±0.09) *	0.71 (±0.08) *	0.21 (±0.09) *
Cow serum total proteins	0.38 (±0.02) *	0.07 (±0.03) *	0.30 (±0.03) *	0.20 (±0.08) *

Phenotypic (σ_p_^2^), additive genetic (σ_a_^2^), residual (σ_r_^2^) variance, and heritability (h^2^) estimates (SE in parentheses). * *p* < 0.05.

**Table 4:** Genetic and phenotypic correlations of cow and calf serum total proteins (%Brix value) with colostrum traits (SE in parentheses).

	Calf Serum Total Proteins		Cow Serum Total Proteins	
	Correlations			
Trait	Genetic	Phenotypic	Genetic	Phenotypic
Colostrum total solids	0.94 (0.14) *	0.33 (0.03) *	0.01 (0.03)	0.05 (0.03)
Colostrum protein content	0.99 (0.14) *	0.31 (0.03) *	0.01 (0.02)	0.04 (0.03)
Colostrum fat content	−0.16 (0.26)	0.04 (0.04)	0.01 (0.01)	0.01 (0.03)
Colostrum lactose content	−0.82 (0.24) *	−0.19 (0.03) *	0.04 (0.03)	−0.10 (0.03) *

* *p* < 0.05.

## Data Availability

Additional data are available on request from the corresponding author.

## Supplementary Materials

The following supporting information can be downloaded at: https://www.mdpi.com/article/10.3390/ani13030366/s1, Text S1: Protocol for the determination of colostrum fat, protein and lactose content with MilkoScan.
