# Supplementary material for: Genetic Parameters of Serum Total Protein Concentration Measured with a Brix Refractometer in Holstein Newborn Calves and Fresh Cows
Source: Animals (Basel). 2023 Jan 20;13(3):366. doi: 10.3390/ani13030366 (PMC9913346; doi:10.3390/ani13030366)
Supplement: Supplementary file 1 [file animals-13-00366-s001.zip › animals-2099663-supplementary.pdf]

S1. Protocol for the determination of colostrum fat, protein and lactose content with MilkoScan

The determination of the colostrum fat, protein and lactose content was performed with an automated analytical device (MilkoScan™ Minor, Foss, Denmark) following the protocol below:

a) Several colostrum samples were analyzed with the reference chemical methods for the determination of fat, protein and lactose content. Colostrum samples analyzed, represented a wide range of variation in the content of each component.

b) The above samples were used to calibrate the automated device according to the ISO 9622-IDF 141-2013 standard following the manufacturers' instructions.

c) Colostrum samples forming our data-base were heated in a water bath at 40±2°C to completely liquefy their fat and mixed gently by inverting at least 10 times to ensure uniform distribution of the components. Then the samples were diluted 1:4 with distilled water and analyzed with MilkoScan.

To evaluate the precision and accuracy of the method, colostrum samples with low fat, protein and lactose content were fortified with homogenized cream, whey protein concentrate and lactose monohydrate at different levels, and analyzed according to the described procedure. The results based on five independent determinations at each fortification level showed that the mean recovery for fat, protein and lactose was 98.5%, 99.2% and 101.4%, respectively, whereas the precision (RSD%) ranged between 3.9% and 6.1%.

The whole process took place at the Laboratory of Safety and Quality of Dairy Foods, School of Veterinary Medicine, Faculty of Health Sciences, Aristotle University of Thessaloniki.
